# Social and Cultural Factors Leading to Suicide Attempt via Organophosphate Poisoning in Nepal

**DOI:** 10.1155/2019/7681309

**Published:** 2019-08-18

**Authors:** Christine Licata, Lawrence Liu, Dale Mole, Jonathon Thorp, Rajesh Chand, Sabin Chaulagain

**Affiliations:** ^1^School of Medicine, Loma Linda University, Loma Linda, USA; ^2^Department of Emergency Medicine, Scheer Memorial Adventist Hospital, Banepa, Nepal; ^3^Department of Internal Medicine, Scheer Memorial Adventist Hospital, Banepa, Nepal; ^4^Department of Social Work, Scheer Memorial Adventist Hospital, Banepa, Nepal

## Abstract

Organophosphates are commonly used in rural, agricultural communities worldwide. Poisoning in Nepal is commonly a result of suicide attempt via ingestion with a mortality rate of 41 times higher than in the United States even after appropriate treatment. The patient discussed in this case is a 46-year-old Nepalese female with a complicated psychosocial history that presented with a suicide attempt via organophosphate ingestion. She required higher doses and greater lengths of treatment of atropine and pralidoxime to resolve symptoms of toxicity that resulted in atropine-induced psychosis, a side effect rarely cited in the literature. This patient was one of many who attempt suicide in Nepal, where suicide is the leading cause of death for young to middle aged women in the country.

## 1. Introduction

In Nepal, most of the cases of organophosphate (OP) toxicity occur as a result of suicide attempt via ingestion by women in agricultural communities whereas, in the United States (US), the cases of OP poisoning (OPP) are the result of accidental dermal exposure and intentional or accidental ingestion by farmers in agricultural areas. The mortality rate for appropriately treated OP poisoning is 7.4% in Nepal, 10% worldwide, and 0.18% in the US [1].

OP insecticides are widely available because most families grow their own produce in their backyard (most common: methyl parathion and dichlorvos; less common: dimethoate, profenofos, and chlorpyrifos) [2]. Within minutes to hours of exposure or ingestion, patients may experience signs and symptoms of cholinergic excess: bradycardia, miosis, lacrimation, salivation, bronchorrhea, and bronchospasm. After 1-4 days of exposure, 40% of patients will develop nicotinic symptoms: fasciculations and bulbar, proximal, and respiratory muscle weakness.

Diagnosis is largely clinical and requires a low index of suspicion to ensure rapid treatment. For questionable diagnoses, an atropine challenge (administer 1 mg IV atropine) can be performed. The absence of signs and symptoms of anticholinergic excess (dry mouth, rise in heart rate > 35 beats per minute within 5 minutes of administration, mydriasis, blurred near vision, and dry, hot skin) supports the diagnosis of OPP. For confirmation, an RBC acetylcholinesterase activity test can be performed. In Nepal, the grading of OP poisoning is based on the Peradeniya Organophosphorus Poisoning Scale (POPS) [3]. POPS utilizes the parameters of pupil size, respiratory rate (RR), heart rate (HR), fasciculations, level of consciousness (LOC), and seizures to rate the poisoning as mild, moderate, or severe. Management should start with immediate respiratory support on suspicion of OPP followed by the placement of personal protective equipment (PPE) and decontamination.

Atropine is started at a dose of 1.8 to 3.0 mg IV bolus for adults with dose doubling every 3 to 5 minutes until resolution of bronchorrhea and wheezing. Pralidoxime 2 g IV is infused over 30 minutes. If seizures develop, diazepam 10 mg IV is administered and repeated until seizures stop. If the lungs are clear to auscultation and blood pressure (BP) and HR are adequate, atropine should be infused at 10 to 20% of the total dose required every hour. After 48 hours, the total daily dose of atropine can be reduced by 20% each day as long as the previously mentioned target signs are maintained. Pralidoxime should be infused at 10 mg/kg/hour for 48 hours [4].

## 2. Case Presentation

The patient is a 46-year-old female with a psychiatric history of Bipolar Disorder I and depression presenting after a suicide attempt via OP ingestion at Scheer Memorial Adventist Hospital in Banepa, Nepal. She ingested 120 mL of chlorpyrifos in 2.5 hours. No one was home at the time. She was found by neighbors who called her estranged daughter to take her to the hospital. The patient reports that her attempt was a result of her daughter marrying a man in a lower caste system, which by Nepalese Hindu culture mandates that the patient disown her.

The patient presented to the emergency department (ED) with bradycardia, miosis, lacrimation, salivation, bronchorrhea, bronchospasm, urination, emesis, and diarrhea. ED vitals were HR of 104 beats per minute (bpm), BP of 140/90 mmHg, oxygen saturation of 98%, temperature of 97°F, RR of 20 breaths per minute. She was immediately given a loading dose of 1.8 mg atropine IV and 2 g pralidoxime IV. Subsequently, charcoal packing and nasogastric lavage were performed within an hour of presentation. She required an additional 2 mg of atropine for resolution of respiratory distress. ED labs were significant for hyponatremia (131 mmol/L) and low serum cholinesterase of (895.5 mU/mL). She was then transferred to the ICU where she stayed for 14 days. She was given 1 g of pralidoxime every 8 hours for 4 days. Her atropine dose was increased 20% per day to maintain HR over 80 bpm until she reached a max dose of 10.2 mg atropine per day (three times the normal dose). The patient's signs and symptoms of cholinergic excess resolved; however, she developed atropine-induced psychosis. Per nursing reports, patient was agitated, delusional, giving inappropriate responses, and alert and oriented to self and day. She was weaned off atropine by 20% each day and by day 8 her psychosis resolved. Her ICU course was also complicated by aspiration pneumonia with fevers (Temperature max of 102.4°F) which resolved with clindamycin. Once she was stable, she was downgraded to a medicine unit where she remained for 4 days and was discharged on hospital day 19.

## 3. Discussion

Scheer Memorial Adventist Hospital serves the people in the district of Kavre, Nepal. During the year of 2018, 145 patients presented at the hospital after a suicide attempt via toxic ingestion with organophosphate poisoning (OPP) being the most common at 41.4%. Of the total suicide attempts, 60% accounted for females. Suicide is currently the leading cause of death for Nepalese women aged 15–49 [5]. There is a gender gap in Nepal when it comes to suicide attempts, with women attempting suicide three times more than men [6].

It is posited that Nepal's high female suicide rate is a result of social and cultural factors that reveal gender inequality in the country. Examples of this inequality include domestic abuse, infidelity of husbands, unwanted pregnancies, and early marriages with the cultural cost of leaving one's family and friends, all of which contribute to the suicide attempts of women in Nepal [7, 8]. Once married, Nepalese women are forced to live with their husband's family and it is culturally unacceptable for them to return to their own family for any reason. This often leaves them feeling trapped with suicide as their only option [9]. For women in particular, the under reporting of suicides and suicide attempts may be caused in part by a “culture of silence,” especially in cases related to domestic abuse [9]. This begs the question to the medical community, how can physicians help prevent suicide when it is so intimately tied to a society's culture?

The caste system in Nepal may have a role in the country's female suicide epidemic. This was discussed with Scheer Memorial Adventist Hospital's clinical social worker who handles all suicide cases. He disclosed that though the government has officially removed the caste system, it is still present in many communities in Nepal. Intermarriage between castes is culturally unacceptable. If a woman marries into a different caste, she is likely to be disowned by her family, and it is common for there to be domestic abuse from the in-laws and husband. In this patient's case, she faced pressure from her husband and eldest son to disown her daughter for marrying below their caste. This conflict and the widespread availability of organophosphate (OP) insecticide were large contributors to her suicide attempt.

This case also highlights a rare adverse effect from the treatment of OPP. As a result of her atropine treatment, the patient developed atropine-induced psychosis, a reaction rarely cited in the literature. Psychotic symptoms such as restlessness, excitement, hallucinations, and delirium have been recorded following atropine administration [10]. Yet atropine is the mainstay treatment for OPP. It is essential to recognize this potential adverse reaction of atropine when prescribing doses of it intravenously. Immediate withdrawal of atropine is necessary to avoid further complications [10]. The medical significance of this case involves the management of OP poisoning after large volume ingestion (120 mL). As noted in the case presentation, this patient required three times the dose of atropine normally needed for standard patient treatment, maxing at a dose of 10.2 mg/day. Ingestion of greater than 101 mL of OP correlates with a mortality rate of 28.6 [11].

This patient's atropine-induced psychosis may have been exacerbated by her underlying psychiatric disorders. In Nepal, mental health disorders are not well understood, and it is possible that this patient's psychiatric disorders had not been adequately treated. In fact, Nepal is a “country where only 1% of the national health care budget is dedicated to mental health.” [9] Nepal exemplifies the global burden of mental health disorders in low income countries. In low income countries, around 80% of the population are unable to seek adequate mental health care and there is an estimated shortage of 1.18 million mental health professionals [12, 13]. Mental health problems account for 7.4% of disability adjusted life years (DALY) and 22.9% of all years lived with disability (YLD) [14]. This is an issue of concern for the medical community on the global scale given the lack of mental healthcare in low income countries.

## 4. Conclusion

This case demonstrates the use of high dose atropine and pralidoxime therapy to effectively treat organophosphate poisoning following large volume ingestion and displays the need for physicians to recognize atropine-induced psychosis. It also highlights the complex nature of the medical community's role in suicide prevention when culture is a contributing factor.
